# Long-term consequences of prenatal saccharin exposure: evidence of sex-specific molecular programing in the prefrontal cortex and behavior of adolescent rats

**DOI:** 10.3389/fnbeh.2026.1815692

**Published:** 2026-04-30

**Authors:** Beatriz Pacheco-Sánchez, Raquel López-Merchán, Pablo Rubio, Pilar García-Martos, Juan Suárez, Carlos Sanjuan, Leticia Rubio, Stella Martín-de-Las-Heras, Fernando Rodríguez de Fonseca, Francisco Alén, Marialuisa de Ceglia, Patricia Rivera

**Affiliations:** 1 Unidad de Gestión Clínica de Salud Mental. Hospital Universitario de Málaga, Málaga, Spain; 2 IBIMA Plataforma Bionand, Instituto de Investigación Biomédica de Málaga, Málaga, Spain; 3 Departamento de Anatomía Humana, Medicina Legal e Historia de la Ciencia, Universidad de Málaga; 4 Euronutra S.L., Málaga, Spain; 5 Unidad de Gestión Clínica de Neurología, Hospital Regional Universitario de Málaga, Málaga, Spain; 6 Departamento de Psicobiología, Facultad de Psicología, Universidad Complutense de Madrid, Madrid, Spain

**Keywords:** endocannabinoid system, GABA, gestational saccharin, glutamate, legal medicine, non-nutritive sweeteners, prefrontal cortex, toxicology

## Abstract

**Introduction:**

Certain events that occur in early life, such as changes in nutrition, can induce structural and functional modifications in brain development, leading to behavioral programing in the offspring. These effects depend on the timing, intensity, and duration of exposure, and may contribute to chronic disorders in adulthood. Artificial non-nutritive sweeteners (NNS), such as saccharin, have recently been proposed as potential developmental disruptors. Saccharin consumption during pregnancy is discouraged, as it can cross the placenta and accumulate in the fetus.

**Methods:**

In this study, pregnant rats were administered 0.1% saccharin in drinking water throughout gestation. On postnatal day 21, offspring were assessed for behavioral outcomes using the open field and elevated plus maze tests. During sacrifice, the prefrontal cortex of the animals was collected.

**Results:**

Gestational saccharin exposure induced sex-specific behavioral changes: offspring of saccharin-consuming mothers spent more time in the center of the arena, while only females showed increased open-arm entries. These alterations were coupled with changes in prefrontal endocannabinoid, glutamatergic, and GABAergic gene expression. Only saccharin-exposed male pups showed significant alterations in Dagla, Daglb and Gpr55 along with increased expression of glutamatergic receptors (*Grin1, Grin2a, Grin2c, Gria1, Grm3*). Females exhibited reduced expression of GABAergic receptor genes (*Gabrg2, Gabbr2*), and significant changes in the phosphorylated expression of proteins involved in the insulin pathway (IRS-1, PI3K, AKT, GSK3b).

**Discussion:**

These findings suggest that developmental NNS exposure produces long-lasting behavioral outcomes in offspring, which are linked to alterations in multiple signaling pathways within the prefrontal cortex.

## 1 Introduction

The study of intrauterine conditions and those of the first years of life (perinatal programing) is vital, considering that exposure to diet, drugs, toxins, and stressors during these moments can expose the offspring to the development of various metabolic, cognitive, and behavioral pathologies. In fact, it is essential to consider that events occurring in the early stages of life, including nutritional alterations during pregnancy, can lead to various structural and functional modifications in neurodevelopment in the short, medium, and long term, depending on the moment, dose, and duration of these events (Cusick and Georgieff, 2016; Matonti et al., 2020; Nelson and Gabard-Durnam, 2020; Rivera et al., 2020).

A controversial issue regards the use of sweeteners during pregnancy, for example. In recent years, there has been more focus on excessive sugar consumption, and as a result, the use of low-calorie sweeteners has increased. Preclinical studies in mice have demonstrated that regular consumption of low doses of artificial sweeteners can alter the activity of brain regions involved in decision-making and reward processes, exerting variations in brain neurochemistry, such as changes in the content and turnover of dopamine, while avoiding metabolic regulation (Bielavska et al., 2000; Hamelin et al., 2022). Admittable ingest of saccharin during pregnancy is of 5 mg/kg/day; however no consumption at all is recommendable. Generally, consumption of artificial sweeteners has been associated with a risk of premature birth, without a linear relation among consumed daily quantities and risk. No increased risk of spontaneous abortions in the fetuses was registered in a case-control study due to pregnancy consumption of saccharin, however is consumption is forbitten in various countries during pregnancy (Cavagnari, 2019). However, the perinatal effect of sweetener consumption on these regions still has to be investigated.

Saccharin is one of the most widely used sweeteners. It has been considered safe for human consumption, although its use is widely debated due to its genotoxicity, with the potential to induce cancer and damage germ cells (Cavagnari, 2019). Consumption of saccharin is discouraged during pregnancy since it can cross the placenta and accumulate in the fetus, potentially programing its metabolism for the rest of life, giving rise to diseases such as obesity, alterations in the microbiota, as well as behavioral disorders such as preferences for sweet taste (Suez et al., 2014; Cavagnari, 2019; Olivier-Van Stichelen et al., 2019). However, the molecular mechanisms involved in this perinatal programing are unknown.

Previous studies from our group demonstrated that saccharin consumption during pregnancy provokes disruptions in the glucose metabolism of the offspring, altering GLP-1 and cannabinoid signaling in both the intestine and the hypothalamus in a sex-dependent manner (Pacheco-Sánchez et al., 2025). However, the possible effects of saccharin consumption on the main signaling pathways of the prefrontal cortex (PFC), the center of executive functioning, closely related to the control of cognitive, behavioral, and neuroendocrine processes, have not yet been delved into (Lesiewska et al., 2022).

The PFC plays a crucial role in food-related decision-making and processing food rewards, as well as in regulating cognition and behavior. The PFC is a brain region with high sensitivity towards insulin, with the latest research suggesting that specific subpopulations of inhibitory neurons may even synthesize insulin locally, thus exerting an effect in local neural circuits (Csajbók and Tamás, 2016). Insulin regulates brain metabolism and energy homeostasis, stimulating glucose uptake and glycolysis, which is essential in memory formation, cognitive tasks and synaptic development (Chiu and Cline, 2010). Moreover, insulin acts as a neuromodulatory peptide, controlling neuronal survival, development and growth (Agrawal et al., 2021). Importantly, insulin also modulates inhibitory neurotransmission by facilitating the trafficking of γ-aminobutyric acid (GABA) receptors to both inhibitory synapses and extra synaptic sites (Agrawal et al., 2021).

In the PFC, maintaining excitation/inhibition balance is critical, as its disruptions are strongly associated with behavioral disorders arising from chronic stress or neural insults (Labouesse et al., 2015). GABA and glutamate maintain the electrical balance of cells, with glutamate serving as the primary excitatory neurotransmitter and GABA as the main inhibitory one in the central nervous system (CNS). Excessive glutamate activity and/or insufficient GABA can lead to the formation of negative associative memories, coupled with an increased risk of emotional dysregulation (Marchisella et al., 2021). Thus, the proper release of these neurotransmitters is vital for an adaptive behavioral response, which is also influenced by cannabinoid signaling.

The endocannabinoid system (ECS) is a lipid-based signaling system, present at both central and peripheral levels, primarily involved in regulating energy metabolism, eating behavior, and energy homeostasis (de Ceglia et al., 2021). Dysregulation of this system has been associated with the development of obesity and neuropsychiatric diseases (McLaughlin et al., 2014; Gatta-Cherifi and Cota, 2015; Maldonado et al., 2020; Joshi and Onaivi, 2021). The ECS comprises cannabinoid receptors, ligands or endocannabinoids, as well as enzymes involved in synthesis and degradation. The two main endocannabinoids are 2-arachidonoylglycerol (2-AG) and anandamide (AEA). 2-AG and AEA are ligands to cannabinoid type 1 and 2 receptors (CB1 and CB2), but they can also activate other types of G-coupled receptors, such as GPR55. The enzymes involved in the synthesis of endocannabinoids are diacylglycerol lipases α and β (DAGLα/β) and N-acyl phosphatidylethanolamine-specific phospholipase D (NAPE-PLD); the enzymes responsible for degradation are Monoacylglycerol lipase (MAGL) and Fatty Acid Amide Hydrolase (FAAH), respectively (de Ceglia et al., 2024).

In the brain, the ECS is highly concentrated in interneuron synapses, playing a critical role in modulating the interactions among various neurotransmitter pathways, including glutamate and GABA. At the same time, the synthesis of endocannabinoids occurs on demand in neurons, depending on the release of GABA or glutamate from presynaptic cells. Following, endocannabinoids act as retrograde neurotransmitters on CB1 and CB2 receptors located on presynaptic glutamatergic or GABAergic neurons, thus modulating the excitation/inhibition balance within a given neuronal circuit (Castillo et al., 2012; Katona and Freund, 2012; Kano, 2014).

Maternal nutrition during gestation can lead to specific changes in the PFC and its role in reward circuits. Interestingly, insulin signaling appears to play a crucial role in perinatal programing, as it is extremely sensitive to maternal metabolic status and nutritional environment. Disruptions in maternal insulin levels, whether due to gestational diabetes, obesity, or malnutrition, may lead to long-term alterations in offspring metabolism, neurodevelopment, and energy homeostasis (Dearden and Ozanne, 2015; Saravanan, 2020; Lippert and Brüning, 2022). Similarly, numerous studies demonstrate the involvement of the endocannabinoid system in the phenomenon of perinatal programing. Recently, it has been suggested that the endocannabinoid signaling pathway may be affected by maternal nutrition; therefore, this system could participate as a molecular substrate for perinatal programing (Ramírez-López et al., 2016; Rivera et al., 2020; Tovar et al., 2021; Rivera et al., 2022).

Previously, we have demonstrated that saccharin consumption during gestation can have an impact on glucose metabolism in the offspring in a sex-dependent manner, through significant alterations to the endocannabinoid and GLP-1 pathway both in the hypothalamus and intestine (Pacheco-Sánchez et al., 2025). In recent years, several studies have reported sex-specific responses in behavior, neurodevelopment, metabolism and brain signaling pathways among others (Chung and Auger, 2013; Varlamov et al., 2014; Gegenhuber and Tollkuhn, 2019, 2020; Iqbal et al., 2024; Melgar-Locatelli et al., 2024), underlying that the study of sex-specific variations is pivotal towards the future of precision medicine and for the full understanding of the biological mechanisms underlying health and disease. With this in mind, we analyzed all study parameters by distinguishing between male and female individuals and performed statistical analyses accordingly to identify potential sex-specific differences in both control animals and those exposed to saccharin. It is important to underscore, however, that the analysis of sex-specific effects in this manuscript is exploratory in nature. Moreover, the specific mechanisms underlying these sex differences, as well as the role of sex or sex hormones in shaping the observed effects, fall beyond the scope of this study and have not been explored in detail.

With this study, we want to build new evidence upon the previous, demonstrating that saccharin consumption during pregnancy not only impacts the metabolism of the offspring but can also have effects on behavior. With this aim, animals underwent OF and EPM tests, which are classically used to assess anxiety-like behavior; however, they are also widely employed to evaluate exploratory activity, risk assessment, and responses to novelty, processes that are closely linked to prefrontal cortex function (Dalley et al., 2004; Crawley, 2008). At the same time, we also evaluated important signaling pathways in the PFC which are associated which are responsible of neuronal survival, excitatory-inhibitory balance and metabolism such as insulin, cannabinoid, glutamatergic, and gabaergic signalings.

## 2 Materials and methods

### 2.1 Ethics statement

All experimental procedures with animals were conducted in strict accordance with the guidelines of Royal Decree 1201/2005 of October 21, 2005 (BOE no252), and by Directive 86/609/ECC of the European Community (November 24, 1986) about the regulation of animal research.

### 2.2 Animals and experimental design

Ten adult female Wistar rats (Charles River Laboratories, Saint-Germain-Nuelles, France) were singly housed in standard cages, with *ad libitum* access to water and food in a temperature- and humidity-controlled room (22 ± 2°C and 55 ± 5%, respectively) on a 12-h light/dark cycle. At the beginning of the experiment, female rats were mated in-house with five males (ratio 1 male: 2 females). After confirming that the rats were pregnant through vaginal plug check detection (day of copulatory plug assessment was then assigned as gestational day 0, GD0), dams were randomly assigned to two groups: saccharin and control. The saccharin group received 0.1% (w/v; 0.1 g in 100 mL) of saccharin (Thermo Scientific, #149005000) during all the gestation until delivery of the pups, diluted in their usual water bottle (this was their only access), while the control group only drank water. No access to saccharin was given to dams during lactation. All the animals were guaranteed *ad libitum* access to standard chow diet (SAFE D04, Scientific Diets), during gestation and lactation. Additional information about initial and pregnancy body weight of dams (measured weekly) and litter size, as well as pups’ weight after birth (PND3) and pups’ sex ratio in each litter are available in Supplementary section 1.1–1.3.

At PND21, the litters were paired, resulting in four groups of 16 individuals each, based on sex and the beverage to which the mothers were exposed during gestation. Between 2 and 4 individuals per litter and sex were included in each experimental group. Half of the animals were randomly assigned to groups for behavioral analysis at weaning (*n* = 8), and the other half were sacrificed for biochemical analysis at the PFC (*n* = 8). A graphical representation of the methodology of the study has been included in Supplementary Figure S1.

### 2.3 Behavioral testing

After weaning (PND21), behavior was evaluated using the open field test (OF; PND22). The day after OF test (PND23), animals underwent Elevated Plus Maze paradigm (EPM), allowing a recovery in-between tests. OF and EPM are classically used to assess anxiety-like behavior, however, they are also widely employed to evaluate exploratory behavior, risk assessment, and responses to novelty, which are closely related to prefrontal cortex function (Dalley et al., 2004; Crawley, 2008). PND21 was chosen as a timepoint for this analysis because it’s the earliest age where pups are independent enough and neurologically mature enough for meaningful behavioral testing while still reflecting early developmental effects.

OF test was performed in an opaque arena (90 × 90 × 40 cm; Panlab, #76-0189) divided into 16 squares with two zones; the center (4 squares) and the periphery (12 squares). The OF was illuminated using a ceiling halogen lamp that was regulated to yield 350 lux at the center of the field. On the experimental day, the animals were placed in the center, and the locomotor activity and time spent in the center and periphery were scored for 5 min (Sanchez-Marin et al., 2017). A video monitor was used to record the animals’ activity during the test. The apparatus was cleaned between trials with 70% ethanol in-between tests.

The EPM apparatus (Panlab, #LE842) consisted of opaque plastic and featured two oppositely positioned open arms (45 × 10 cm), two closed arms of the same size, and walls 50 cm high. These arms connected via a central neutral area (10 × 10 cm). The entire apparatus was elevated 75 cm above a white floor and illuminated at 70 lux. Initially, the rats were placed in the center of the maze, facing an open arm, and were allowed to explore freely for 5 min. The number of entries (an arm entry was defined as all four paws in the arm zone) and the time spent in each arm were recorded using a video monitor. The apparatus was cleaned between trials with 70% ethanol in-between tests.

Both OF and EPM were recorded using a video camara. *A posteriori*, the videos were analyzed with EthoVision XT software (Noldus Information Technology, Wageningen, The Netherlands). Distance traveled in both tests was measured in cm; time is expressed in seconds (s).

### 2.4 Sample collection

The pups were sacrificed a day after (post-natal day 24). The animals were anesthetized with pentobarbital (50 mg/kg) and sacrificed by decapitation. Pentobarbital was administered prior to decapitation to ensure a humane and ethically acceptable procedure. As a potent central nervous system depressant, pentobarbital rapidly induces deep anesthesia, rendering the animal unconscious and insensitive to pain. This approach minimizes stress and distress while ensuring compliance with established animal welfare guidelines. Additionally, reducing pre-mortem stress helps to limit potential alterations in physiological and biochemical parameters, thereby improving the reliability of experimental outcomes. Brains for dissection and biochemical analysis (*n* = 8 for each group) were extracted and stored at –80°C. After freezing, the prefrontal cortex (PFC) was dissected by cutting from + 4.7 mm to + 2.2 mm according to the Bregma coordinates following the Paxinos atlas (Paxinos and Charles, 2007). On the other hand, brains from animals which underwent behavioral tests (*n* = 8 for each group) were extracted and fixed in a solution of paraformaldehyde 4% (Santa Cruz, SC-281692) and then stored at -20. Then, they were cut into coronal sections of 30 micrometers using a vibratome; sections were then stored in PBS + 0.01% Sodium Azide at 4°C until inmuno staining.

### 2.5 RNA isolation and real-time quantitative PCR analysis

Real-time qPCR was performed similarly to Ruiz-Viroga et al. (2022) and Tovar et al. (2024) using specific sets of TaqMan Gene Expression Assays (ThermoFisher Scientific; Supplementary Table 1). RNA from the left part of the PFC (*n* = 8) was extracted using the Trizol method according to the manufacturer’s instructions (ThermoFisher Scientific, #15596018). Briefly, 50–100 mg of tissue was homogenized in 1 mL of TRIzol reagent. For samples with high lipid content, lysates were centrifuged (12,000 × g, 5 min, 4–10°C), and the clear supernatant was transferred to a new tube. After a 5 min incubation at room temperature, 0.2 mL of chloroform per 1 mL of TRIzol was added, followed by vigorous mixing and incubation for 2–3 min. Samples were centrifuged at 12,000 × g for 15 min at 4°C to separate phases, and the aqueous phase was carefully collected. RNA was precipitated by adding 0.5 mL of isopropanol per 1 mL of TRIzol used, incubated for 10 min at room temperature, and centrifuged at 12,000 × g for 10 min at 4°C. The RNA pellet was washed with 75% ethanol, centrifuged at 7,500 × g for 5 min at 4°C, air-dried, and resuspended in RNase-free water. The samples were incubated at 55–60°C for 10–15 min to facilitate complete dissolution. RNA concentration and purity were assisted calculating the A260/A280 ratio using a Nanodrop Spectrophotometer (ThermoScientific). 1 μg of RNA was then retrotranscripted into cDNA using iScript™ Reverse Transcription Supermix (BioRad; #1708841) following manufacturers’ instructions (5 min at 25°C; 20 min at 46°C; 1 min at 95°C). qPCR analysis was run on a CFX-Duet Real-Time PCR System (Bio-Rad), with the following protocol (2 min at 50°C; 10 min at 95°C; 44 cycles of 15 s at 95°C and 1 min at 60°C; 10 min at 20°C). For each gene evaluated, a melting curve analysis was performed to ensure that only a single product was amplified. After analyzing several control genes, values obtained from the samples were normalized to beta actin (*Actb*) levels, which did not vary significantly between groups (two-way ANOVA, no significant effect of sex, treatment and interaction F respectively 0.9080; 0.06754 and 0.7952; p respectively 0.3488; 0.7969; 0.3801). β-actin is a widely expressed cytoskeletal protein whose expression is consistent in neuronal and glial populations of the prefrontal cortex, allowing it to serve as an internal control, enabling accurate quantification of target gene expression while minimizing variability due to RNA input or reverse transcription efficiency.

Relative gene expression was calculated using the 2^(−ΔΔCt) method, and represented as fold-change toward male control group, whose values of expression were arbitrary set as 1.

### 2.6 Western blot analysis

Western blotting was performed as described previously (Galeano et al., 2023). Briefly, the right part of prefrontal cortex samples (*n* = 6) were homogenized in 500 μL of ice-cold lysis buffer containing 10% Triton X-100 (ThermoScientific, #A16046AP), 1 mM ethylenediaminetetraacetic acid (EDTA) (Panreac, #131669.1209), leupeptin (ChemCruz, #SC-215243A), sodium fluoride (NaF) (Fluka BioChemika, #71519), sodium orthovanadate (NaOV_4_) (Sigma, #S6508-106), protease (Millipore, #535140) and phosphatase inhibitors (Millipore, #524627) using a tissue-lyser system (TissueLyser II, Qiagen). After centrifuging at 15,000 × g for 15 min at 4°C, the supernatant was transferred to a new tube. The Bradford method was used to measure the protein concentration of the samples (Coomassie protein Assay Reagent, ThermoFisher, 1856209, protocol as per manufacturer’s instructions).

A quantity of 30 μg of each total protein sample was separated into 4–12% polyacrylamide gradient gels (commercially pre-cast; Biorad, #3450124) in MES buffer (Biorad, #1610789) during 3 h at 150 V (Biorad; PowerPac HC Power Supply, #1645052). A molecular weight marker was included in each gel (Amersham™ ECL rainbow molecular weight marker #RPN755E). The gels were then transferred onto nitrocellulose membranes during 1 h at 80 V (Bio-Rad, #1620112) in transfer buffer [0.25 M Tris (Biorad, #1610716), 1.2 M glycine (Biorad, #1610717) and 20%v/v methanol(Millipore, #1060091000)] and stained with Ponceau red. Membranes were blocked in TBS-T (50 mM Tris-HCl (Biorad, #1610716), pH 7.6, 200 mM NaCl (Supelco, #1064041000), and 0.1% Tween 20 (Sigma Aldrich, #102700386) with 2% albumin fraction V from bovine serum (BSA, Roche, Mannheim, Germany) for 1 h at room temperature. The primary antibodies to the proteins of interest (Supplementary Table 2) were incubated overnight at 4°C in a solution of TBS-T with 2% albumin. Mouse Adaptin was used as the reference protein. Adaptin is a component of the clathrin-mediated vesicle trafficking machinery and is expressed at relatively constant levels in neuronal and glial cells. Its stable expression in the PFC makes it suitable as a loading control for Western blotting, ensuring that observed differences in target protein levels reflect true biological variation rather than differences in protein loading or transfer efficiency. After several washes in TBS-T (at least 3 of 10 min of duration), an HRP-conjugated anti-rabbit or anti-mouse IgG (H + L) secondary antibody (Promega, Madison, WI, USA; rabbit #W401B, mouse #W402B), diluted 1:10,000 in a solution of TBS-T with 2% albumine, was added, followed by incubation for 1 h at room temperature. Each membrane was incubated only with its corresponding secondary antibody. After extensive washing in TBS-T (at least 3 of 10 min of duration), the membranes were incubated for 1 min with the Clarity Max Western ECL Substrate (Bio-Rad, United States), and the specific protein bands were visualized and quantified by chemiluminescence using a ChemiDoc*™* MP Imaging System (Bio-Rad, United States). Densitometric analysis of bands was run using ImageJ software (NIH, Bethesda, MD, United States). The results are expressed as the target protein/Adaptin ratios and represented as fold-change toward male control group, whose values of expression were arbitrary set as 1. For the analysis of total and phosphorylated proteins, and to prevent cross-reactivity, membranes were stripped between successive incubations using a solution of 2% SDS (Biorad, #1610302), 62.5 mM Tris HCL, 100 mM betamercaptoethanol (Sigma, #M3148) during 30 min at 50°C. After stripping, membranes were washed 6 times during 30 min in TBS-T and blocked during 1 h with 2% BSA before successive incubation.

### 2.7 Statistical analysis

Data were expressed as the mean ± standard error of the mean (Standard Error Mean, SEM). Parametric analyses were conducted using one- or two-way ANOVA, as appropriate. Although formal tests for normality (e.g., Shapiro–Wilk) and homogeneity of variance (e.g., Levene’s or Brown–Forsythe tests) were not performed, sample sizes were balanced across groups and of moderate magnitude, and visual inspection of the data did not reveal extreme skewness or outliers. Classical literature and simulation studies indicate that ANOVA is robust to moderate deviations from normality when group sizes are comparable and variances are similar (Blanca et al., 2017). Based on these considerations, standard parametric ANOVA was applied, and all reported *p*-values are two-tailed. For outlier identification, the ROUT (Robust Regression and Outlier Removal) method was applied, with a Q value set at 5%, which controls the maximum false discovery rate. Statistical analysis of these data was performed using a two-way ANOVA (factors: sex and saccharin) with GraphPad Prism 8.0.2 and SPSS software. Subsequently, multiple comparisons were made between the different groups using *post-hoc* tests. In those cases where there was an effect of sex, saccharin or both, without any effect of interaction, a simple effects analysis (Fisher’s test) was used. In the cases where no effect of ANOVA was detected or a significant effect of interaction was shown, the Tukey test was applied. A *P* < 0.05 was considered statistically significant. Supplementary Tables 3–8 explain which *post-hoc* test was applied for any analysis. In the present study, individual offspring were used as the experimental unit because behavioral and biochemical analyses were performed at the individual level, and variability within the same litter was of interest for the aims of the study. Moreover, animals from different litters were included in each experimental group to minimize potential litter-specific effects.

### 2.8 Brdu staining in the hippocampus

Sections were first washed in Tris-PBS and endogenous peroxidase activity was blocked using 10% H_2_O_2_ in methanol. DNA denaturation was achieved by incubation in 2 N HCl at 37°C, followed by neutralization in borate buffer (0.15 M, pH 8.5). After washes, sections were preincubated in a blocking solution containing 10% goat serum and 0.3% Triton X-100. Sections were then incubated overnight at room temperature with a mouse anti-BrdU primary antibody (1:1,000; e.g., Sigma-Aldrich, Cat# B2531). After washing, sections were incubated with a biotinylated goat anti-mouse secondary antibody (1:500; e.g., Vector Laboratories, Cat# BA-9200) for 2 h, followed by incubation with avidin–peroxidase complex (ExtrAvidin^®^, Sigma-Aldrich, Cat# E2886; 1:1,000). Immunoreactivity was visualized using DAB with nickel enhancement. The reaction was initiated by adding H_2_O_2_ and monitored under visual control. Finally, sections were mounted on slides, dried, dehydrated, and coverslipped. Results in Supplementary material.

## 3 Results

### 3.1 Behavioral tests

Animals underwent open field (OF) and elevated plus maze (EPM) tests to evaluate their behavior.

**FIGURE 1:**
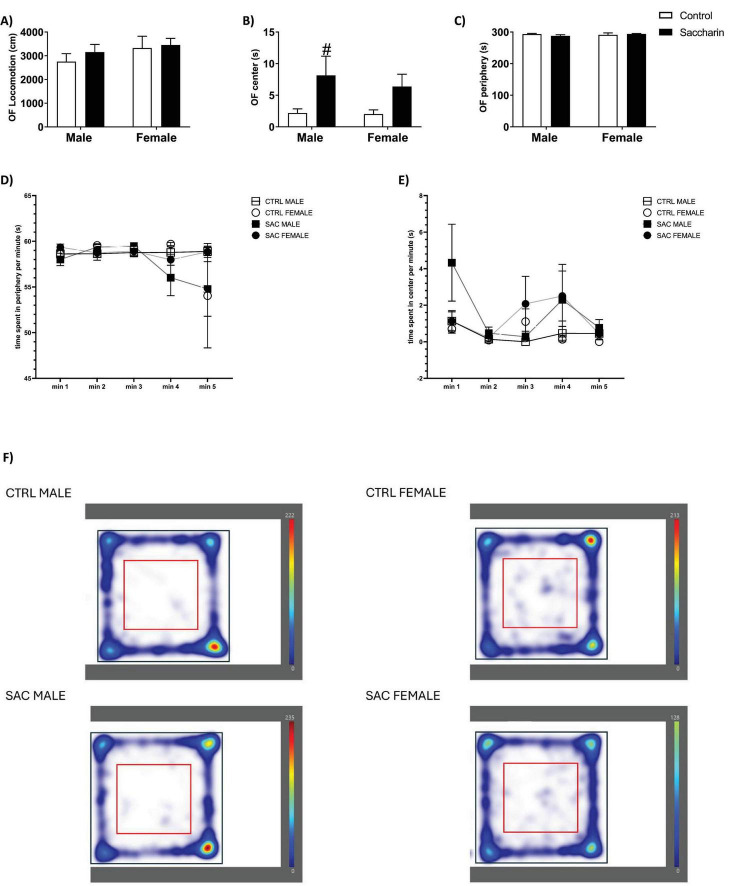
Behavioral characterization of the offspring. Open field (OF) test: **(A)** Total locomotion; **(B)** time spent in the center of the arena; **(C)** time spent in the periphery of the arena; **(D)** time spent in the periphery during the 5 min session; **(E)** time spent in the center during the 5 min session; **(F)** Representative heatmaps of cumulative time per group in OF, elaborated by Ethovision software. In heatmaps, color scale indicates time spent (red = higher occupancy, blue = lower). Results are shown as MEAN ± SEM and analyzed by Two-way ANOVA **(A–C)** or two-way ANOVA for repeated measures **(D–E)**: #*p* < 0.05 vs. control male. *n* = 8 for each experimental group.

In the OF test (Figures 1A–F), we detected no significant difference in the total distance run by the offspring. However, a significant effect of saccharin was detected in the time spent in the center of the arena, being significant in the male offspring when compared to their control group (Fisher *post-hoc*). Thus, both male and female offspring of saccharin-consuming mothers spent more time in the center compared to their control littermates. No significant difference was noticed in the time spent on the periphery. Moreover, when analyzing the per-minute behavior of the pups, ANOVA for repeated measures evidenced a significant effect of saccharin in the time spent exploring the center. In particular, saccharin male offspring spent the highest time exploring the center in the first and fourth minutes when compared to control littermates. Differently, compared to controls, saccharin female offspring spent more time in the periphery during the last minute and more time exploring the center in minutes 3 and 4.

**FIGURE 2:**
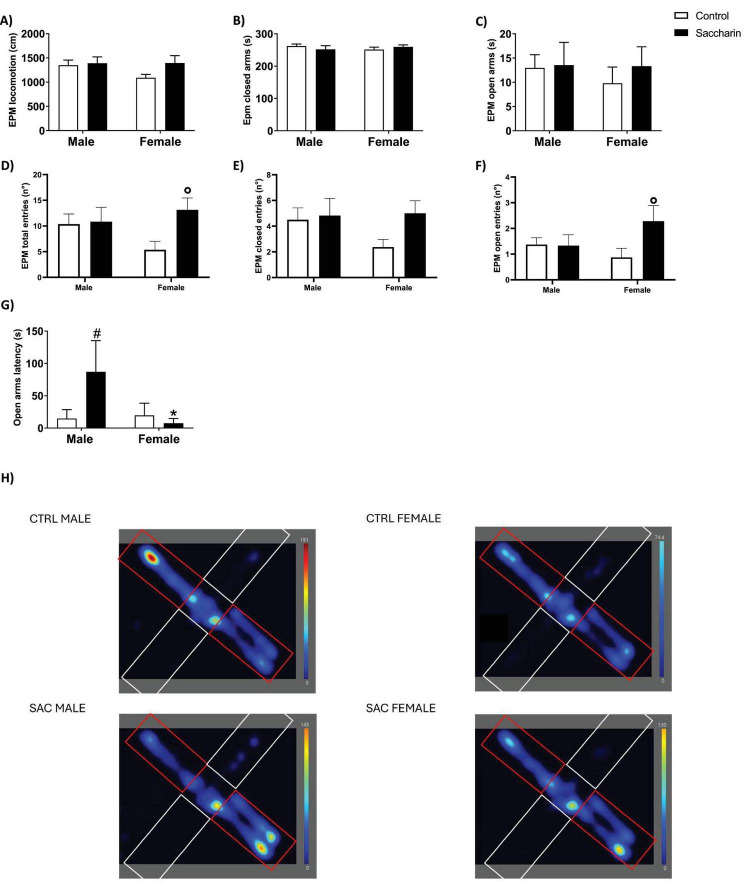
Behavioral characterization of the offspring. Elevated Plus Maze (EPM) test: **(A)** Total locomotion; **(B)** time spent in closed arms; **(C)** time spent in open arms; **(D)** total entries; **(E)** closed arms entries; **(F)** open arms entries; **(G)** latency to first entry in the open arm; **(H)** representative heatmaps of cumulative time in EPM; closed arms in red. In heatmaps, color scale indicates time spent (red = higher occupancy; blue = lower). Results are shown as MEAN ± SEM and analyzed by Two-way ANOVA with Tukey’s *post-hoc*: #*p* < 0.05 vs. control male; *p < 0.05 vs. saccharin male; °*p* < 0.05 vs. control female. *n* = 8 for each experimental group.

In EPM (Figures 2A–H), once more, we detected no significant difference in the total distance run by the offspring. No significant difference was noticed in the time spent in closed or open arms. Female offspring of saccharin-consuming mothers displayed a significantly higher number of entrances (total and open) when compared to the control (significant Tukey’s *post-hoc*; respectively *p* = 0.015 and *p* = 0.023). On the other hand, male offspring of saccharin-consuming mothers showed a significant increase in the latency to enter the open arm when compared to both control littermates and female saccharin offspring (significant Tukey’s *post-hoc*; respectively *p* = 0.047 and *p* = 0.035).

Details of two-way ANOVA analysis are shown in Supplementary Tables 3, 4.

### 3.2 Effect of prenatal saccharin exposure on insulin signaling in the prefrontal cortex of the offspring

**FIGURE 3:**
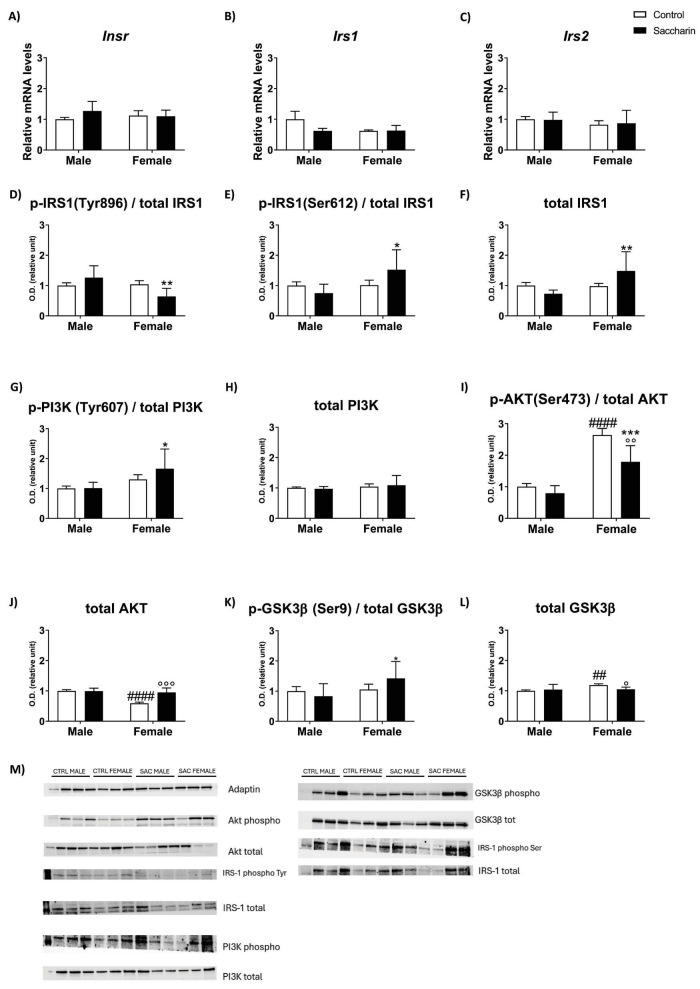
Expression of genes and proteins involved in the insulin pathway and their activation/inactivation by phosphorylation. Genetic expression of: **(A)** Insr, **(B)** Irs1, **(C)** Irs2. Protein expression of: **(D)** p-IRS1Tyr896), **(E)** p-IRS1(Ser612), **(F)** total IRS-1, **(G)** p-PI3K (Tyr607), **(H)** total PI3K, **(I)** p-AKT (Ser473), **(J)** total AKT, **(K)** p-GSK3β(Ser9), **(L)** total GSK3β. **(M)** Significant images of western blot bands. O.D. stands for optical densitometry, measured with ImageJ. Results are shown as MEAN ± SEM and analyzed by Two-way ANOVA: ##*p* < 0.01, ####*p* < 0.0001 vs. control male; *p < 0.05, **p < 0.01, ***p < 0.001 vs. saccharin male; °*p* < 0.05, °°°*p* < 0.01 vs. control female. **(A–C)** *n* = 8 for each experimental group; **(D–L)** *n* = 6 for each experimental group.

Two-way ANOVA indicated that there were no effects of analyzed factors (prenatal saccharin and sex) on the mRNA levels of genes implicated in insulin signaling (*Insr, Irs1, Irs2*) (Figures 3A–C).

Differently, an effect of sex and interaction was shown for the protein expression of pIRS-1(Tyr896), pIRS-1(Ser612), and total IRS-1. Tukey *post-hoc* evidenced a significant decrease in pIRS-1(Tyr896) and a significant increase in pIRS-1(Ser612) and total IRS-1 were noticed in saccharin female offspring, compared to saccharin males (Figures 3D–F).

A sex effect was also found in the protein level of p-PI3K-p85(Tyr607), with the saccharin female group expressing significantly more p-PI3K-p85(Tyr607) than the male group (Fisher *post-hoc*). No significant effect was evidenced by two-way ANOVA in the expression of total PI3K (Figures 3G, H, M).

The main effects of sex and prenatal saccharin were found on the protein levels of p-AKT(Ser473), with females showing higher levels of p-AKT(Ser473) than males regardless saccharin gestational exposure; and prenatal saccharin decreasing p-AKT(Ser473) protein levels in female offspring compared to the female control group (Fisher *post-hoc*; Figures 3I, M).

Two-way ANOVA also revealed the effects of sex, prenatal saccharin exposure, and the interaction between these factors on the protein levels of total AKT, with an increase in AKT expression in the PFC of female offspring born to mothers who consumed saccharin during pregnancy compared to control females (Tukey *post-hoc*). Additionally, a significant decrease in total AKT was observed in control females compared to control males (Figures 3J, M).

Two-way ANOVA evidenced a significant increase in pGSK3β(Ser9) in female offspring of saccharin-consuming mothers when compared to males with the same prenatal conditions (significant effect of sex; Fisher *post-hoc*). A sex effect was also found in the protein levels of total GSK3β. Fisher *post-hoc* analysis showed higher levels of GSK3b in the PFC of control females than males and prenatal saccharin decreasing total GSK3b protein levels in female offspring compared to the female control group (Figures 3K–M).

Details of two-way ANOVA analysis (p, F, degrees of freedom) are shown in Supplementary Table 5 and Supplementary Figure S2 shows Ponceau Red and constitutive bands for each gel.

### 3.3 Effect of prenatal saccharin exposure on the expression of the main genes of the endocannabinoid system in the prefrontal cortex of the offspring

**FIGURE 4:**
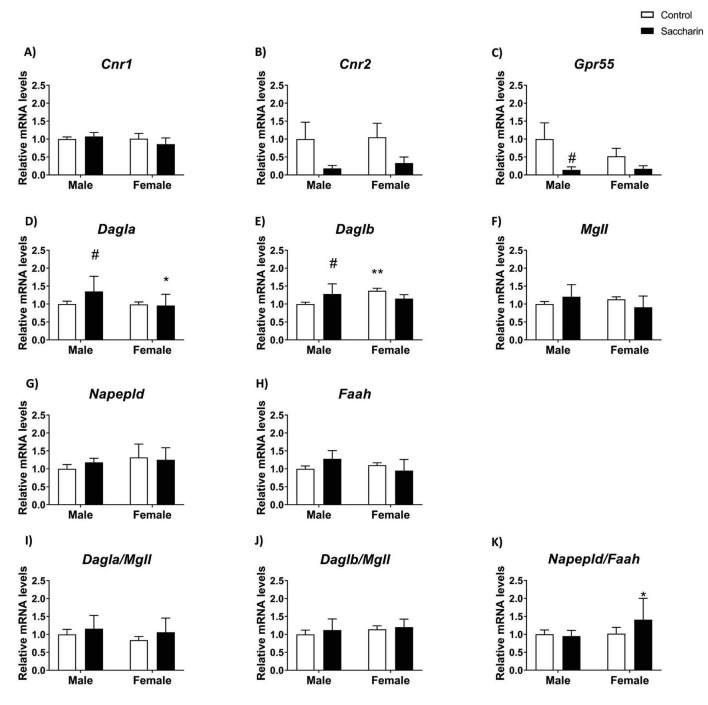
Expression of genes partaking to the endocannabinoid system evaluated by qPCR. Expression of: **(A)** *Cnr1*; **(B)** *Cnr2*; **(C)** *Gpr55*; **(D)** *Dagla*; **(E)** *Daglb*; F) *Magl*; **(G)** *Napepld;***(H)** *Faah*; **(I)** ratio *Dagla/Magl*; **(J)** ratio *Daglb/Magl*; **(K)** ratio *Napepld/Faah*. Results are shown as MEAN ± SEM and analyzed by Two-way ANOVA: #*p* < 0.05 vs. control male; *p < 0.05, **p < 0.01 vs. saccharin male. *n* = 8 for each experimental group.

No effect was noticed for saccharin or sex on *Cnr1* expression (Figure 4A).

Two-way ANOVA indicated a prenatal saccharin effect on *Cnr2* and *Gpr55* mRNA levels, with an overall decrease in *Cnr2* and *Gpr55* expression in animals born to mothers who consumed saccharin during gestation (Fisher *post-hoc*; Figures 4B, C).

An interaction between sex and prenatal saccharin was found on *Daglb*, *Mgll*, and *Faah* mRNA levels, with control females showing higher *Daglb* levels than males. Tukey *post-hoc* analyses also showed increased *Dagla* and *Daglb* expression in the PFC of male offspring born to saccharin-treated mothers compared to the control male group (Figures 4D, E). No increase in *Dagla* and *Daglb* was noticed in the female saccharin group, evidencing a sex-dependent effect. No differences were found between groups using Tukey’s multiple comparisons test for *Mgll, Napepld* and *Faah* expression (Figures 4F–H).

Ratios *Dagla/Magl*, *Daglb/Magl*, *Napepld/Faah* were calculated as an index of synthesis/degradation balance. A significant Tukey *post-hoc* was noticed in the ratio *Napepd/Faah*, with an overall increase in saccharin female rats compared to control female ones (Figure 4K). No ANOVA effects or *post-hoc* differences were found in the *Dagla/Magl* and *Daglb/Magl* ratios (Figures 4I, J)

Details of two-way ANOVA analysis (*p*, F, degrees of freedom) are shown in Supplementary Table 6.

### 3.4 Effect of prenatal saccharin exposure on the expression of the main genes of the glutamatergic system in the prefrontal cortex of the offspring

**FIGURE 5:**
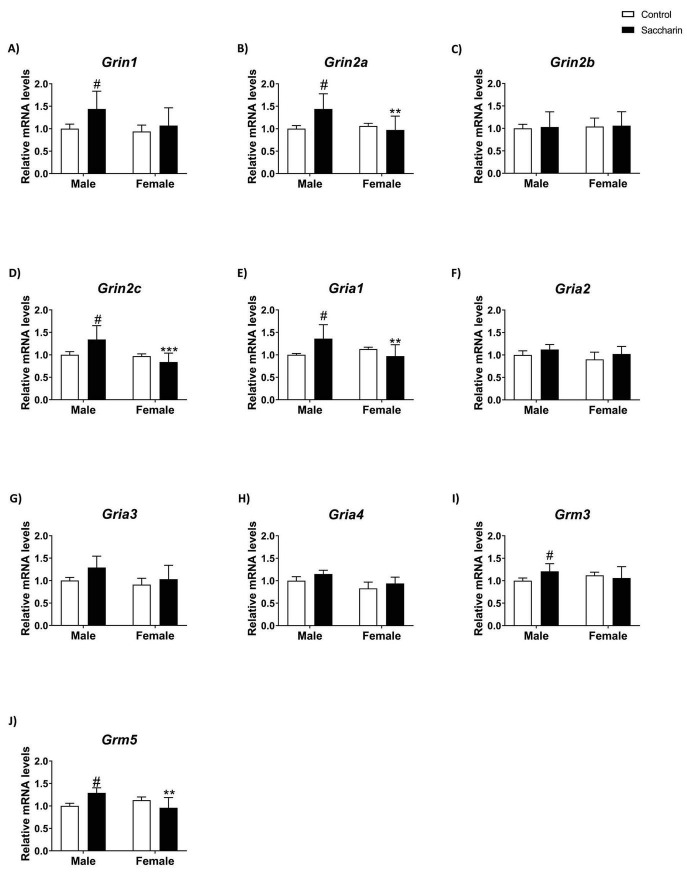
Expression of genes involved in the glutamatergic system evaluated by qPCR. Expression of: **(A)** *Grin1*; **(B)** *Grin2a*; **(C)** *Grin2b*; **(D)** *Grin2c*; **(E)** *Gria1*; **(F)** *Gria2*; **(G)** *Gria3;* **(H)** *Gria4*; **(I)** *Grm3*; **(J)** *Grm5*. Results are shown as MEAN ± SEM and analyzed by Two-way ANOVA: #*p* < 0.05 vs. CTRL MALE; **p < 0.01, ***p < 0.001 vs. SACCHARIN MALE. *n* = 8 for each experimental group.

A treatment effect on *Grin1* mRNA expression was found. Uncorrected Fisher’s LSD *post-hoc* test showed higher *Grin1* mRNA levels in saccharin male PFC compared to the male control group (Figure 5A).

We also found a sex effect and an interaction between sex and prenatal saccharin on *Grin2a* and *Grin2c* mRNA expression, with an overall increase in *Grin2a* and *Grin2c* expression in male offspring born to mothers who consumed saccharin during gestation compared to control and female groups (Tukey *post-hoc*; Figures 5B, D). No significant effect was shown for *Grin2b* mRNA expression (Figure 5C).

Two-way ANOVA revealed an interaction between sex and prenatal saccharin exposure on *Gria1* mRNA expression, with an overall increase in *Gria1* expression in male offspring born to mothers who consumed saccharin during gestation compared to the control and female groups (Tukey *post-hoc*; Figure 5E). No significant variations were noticed in *Gria2* and *Gria3* mRNA expressions (Figures 5F, G). A sex effect on *Gria4* mRNA expression was observed, but no differences were found between groups (Fisher *post-hoc*; Figure 5H).

A significant *post-hoc* analysis was detected for *Grm3* expression, with a significant increase in saccharin male mice compared to control littermates of the same sex (Tukey *post-hoc*; Figure 5I).

An interaction between the two factors analyzed was also found in the *Grm5* expression. Tukey *post-hoc* analysis indicated increased *Grm5* mRNA levels in the PFC of male offspring from mothers who consumed saccharin compared to both control and saccharin females (Figure 5J).

Details of two-way ANOVA analysis (*p*, F, degrees of freedom) are shown in Supplementary Table 7.

### 3.5 Effect of prenatal saccharin exposure on the expression of the main genes of the GABAergic system in the prefrontal cortex of the offspring

**FIGURE 6:**
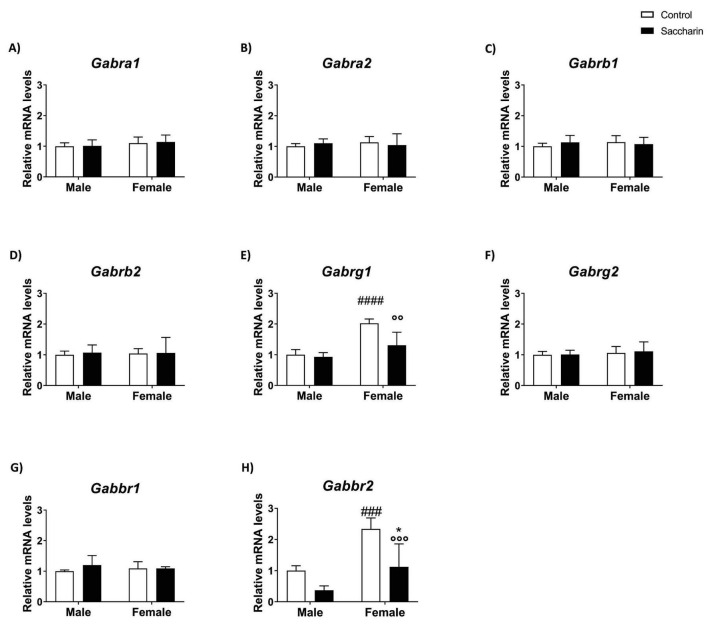
Expression of genes involved in the GABAergic system evaluated by qPCR. Expression of: **(A)** *Gabra1*; **(B)** *Gabra2*; **(C)** *Gabrb1*; **(D)** *Gabrb2*; **(E)** *Gabrg1*; **(F)** *Gabrg2*; **(G)** *Gabbr1;* **(H)** *Gabbr2*. Results are shown as MEAN ± SEM and analyzed by Two-way ANOVA: ###*p* < 0.001, ####*p* < 0.0001 vs. CONTROL MALE; *p < 0.05 vs. SACCHARIN MALE; °°*p* < 0.01, °°°*p* < 0.001 vs. CTRL FEMALE. *n* = 8 for each experimental group.

No significant difference was shown in the expression of *Gabra1*, *Gabra2*, *Gabrb1*, *Gabrb2* (Figures 6A–D).

Two-way ANOVA revealed saccharin and sex effects, as well as an interaction between sex and saccharin, on *Gabrg1* mRNA expression. Tukey *post-hoc* analysis indicated an increase in *Gabrg1* expression in the PFC of control female offspring compared to control male offspring and a significant decrease due to gestational saccharin exposure (Figure 6E). No significant effect was noticed in the expression of *Gabrg2* and *Gabbr1* (Figures 6F, G).

We also found sex and prenatal saccharin effects on *Gabbr2* mRNA expression, with control female offspring showing higher *Gabbr2* mRNA expression than males. Fisher’s LSD *post-hoc* test also showed lower *Gabbr2* mRNA level in saccharin female PFC compared to the control and male saccharin groups (Figure 6H).

Details of two-way ANOVA analysis (*p*, F, degrees of freedom) are shown in Supplementary Table 8.

## 4 Discussion

The present study demonstrates, for the first time, that gestational exposure to saccharin induces changes in the main neurotransmission pathways of the prefrontal cortex in the offspring, which are accompanied by sex-specific alterations in their behavior. Briefly, offspring of saccharin-consuming mothers spent more time in the center of the arena, while only females showed increased open-arm entries. Only saccharin-exposed male pups showed significant alterations in *Dagla*, *Daglb* and *Gpr55*, along with increased expression of glutamatergic receptors (*Grin1, Grin2a, Grin2c, Gria1, Grm3*). Females exhibited reduced expression of GABAergic receptor genes (*Gabrg2, Gabbr2*) and significant changes were observed in the phosphorylated and total expression of proteins involved in the insulin pathway (IRS-1, PI3K, AKT, GSK3β).

### 4.1 Behavioral findings

The results of the behavioral tests suggest that saccharin offspring demonstrate a not-neurotypical behavior. Unlike what we expected, saccharin offspring did not display any sign of anxiogenic behavior, normally associated with increased time spent in the periphery in the OF test or increased time in the closed arms in the EPM. In contrast, both male and female offspring of mothers that consumed saccharin spent more time exploring the center of the open field and exhibited altered patterns of exploration throughout the test compared to their control littermates. Similar behavioral patterns have been reported in spontaneously hypertensive rats (SHR) (Ueno et al., 2002; Pandolfo et al., 2007), which are commonly used as a model of Attention-Deficit/Hyperactivity Disorder (ADHD). This suggests that saccharin-exposed offspring may display behavioral traits reminiscent of SHR rats, potentially reflecting changes in attention, impulsivity, or hyperactivity. Results obtained in the EPM confirm this hypothesis, at least in females: saccharin offspring showed increased total and open arm entries compared to control littermates of the same sex. Arm entries have been suggested to relate to decision-making and/or risk assessment (File, 2001) and, specifically, increased open arm entries have been observed once more in SHR rats or other models of ADHD (Ueno et al., 2002; Pandolfo et al., 2007; Orito et al., 2009; Sterley et al., 2011; Mitchell et al., 2012; Yaoita et al., 2019; de Santana Souza et al., 2020). Not a lot of evidence is available about non-nutritive sweeteners, gestational exposure and behavioral outcomes in the offspring: memory and learning deficits were noticed in the offspring of dams consuming sucrose, stevia (de la Garza et al., 2021), and fructose (Thompson and DeBosch, 2021) during pregnancy; but no evaluation was found about anxious/depressive behavior or attention, impulsivity and risk assessment. On the other hand, similar behavioral results to the ones shown in this study were obtained with gestational exposure to stress (Holubová-Kroupová and Šlamberová, 2021) or known endocrine disruptors such as diazinon (Hawkey et al., 2020) or nicotine (Zhang et al., 2018; Mamiya et al., 2020).

Changes in behavior may be related to impaired neuron proliferation. Major exploration of the center in open field and open arms in EPM is typically associated with reduced anxiety, but in this context, it can be interpreted as an alteration in exploratory behavior, response to novelty and risk assessment. Increased exploration of these areas could be interpreted as a less organized and adaptive behavior, a consequence of hippocampal dysfunction (Bannerman et al., 2004; Anacker and Hen, 2017). Supplementary Figures S5, S6 showing impaired BrdU staining in this region may support this hypothesis.

The alterations noticed in the behavior of the offspring exposed to gestational saccharin were coupled with sex-specific disruptions in insulin, endocannabinoid, glutamatergic, and GABAergic signaling in the prefrontal cortex.

### 4.2 Alterations in insulin signaling

The prefrontal cortex is an insulin-sensitive region since it has numerous receptors for this hormone (Lee et al., 2016). Insulin plays a crucial role in neurodevelopment, influencing neurogenesis and neuronal survival, synaptogenesis and plasticity, myelination, and inflammation (Derakhshan and Toth, 2013; Liu et al., 2014; Lee et al., 2016; Chen et al., 2022). In the offspring, sex-specific effects of saccharin consumption during pregnancy were observed in the activation of proteins involved in the insulin pathway. No effect was detected in males, whereas female offspring of saccharin-consuming mothers exhibited signs of insulin resistance in this region, characterized by decreased IRS-1 and AKT activity and decreased GSK3β activation. This evidence is consistent with previous results published by our group, demonstrating alterations in glucose tolerance and insulin secretion following gestational saccharin exposure, as well as similar alterations in the insulin pathway in the hypothalamus (Pacheco-Sánchez et al., 2025). Insulin resistance can impair several neurotransmitters, such as dopamine (Kleinridders et al., 2015), serotonin (Martin et al., 2024), but most importantly glutamate (Pomytkin and Pinelis, 2021) and GABA (de Bartolomeis et al., 2023), which may be responsible for the alterations in behavior. Previously, other modifications in the behavior have been associated with insulin resistance, such as autism (Stern, 2011), schizophrenia (Agarwal et al., 2020), and ADHD (Hsu et al., 2022; Anthony et al., 2024).

### 4.3 Alterations in endocannabinoid signaling

Consistent with our previous findings (Pacheco-Sánchez et al., 2025), gestational saccharin exposure affected the expression of the endocannabinoid system in the offspring, with both male and female rats exhibiting a significant decrease in CB2 and GRP55 gene expression in the prefrontal cortex. The CB2 receptor plays a significant role in neuronal plasticity, regulating neuron firing activity (Jordan and Xi, 2019) and neurotransmitter release (Ferranti and Foster, 2022). Lower expression of this receptor may impact neuronal survival and neurotransmission. Deletion of CB2 can increase baseline locomotion, rearing, and stereotypic movements (García-Gutiérrez et al., 2012) and chronic administration of antagonists in this region results in anxiolytic-like effects, which may explain the variations noticed in the behavior of saccharin rats (García-Gutiérrez et al., 2012). Previously, lower CB2 activity in the cortex was associated with schizophrenia (Szücs et al., 2016) and selective KO of CB2 in dopaminergic neurons produced ADHD-like responses (Canseco-Alba et al., 2022). Literature suggests that GPR55 also modulates neuronal growth (Cherif et al., 2015) and neurotransmission, acting mainly on glutamate (Marichal-Cancino et al., 2017). Not a lot of information is available about GPR55’s role in the prefrontal cortex; however, decreased expression of this receptor was detected in the same brain region of a preclinical model of autism (Kerr et al., 2013), and various evidence suggests a role for GPR55 in regulating emotional behavior (Marichal-Cancino et al., 2017). Consistent with previous data, the expression of CB2 and GPR55 can be influenced by disruptions in the insulin pathway and viceversa, suggesting that the changes observed in both systems may be interconnected through shared signaling cascades affecting glucose metabolism and neurotransmission (Meadows et al., 2016; Youssef et al., 2019). Sex-specific alterations were noticed in the expression of the enzyme Daglb, which was increased selectively in saccharin male offspring when compared to controls. Postsynaptic DAGL controls neuronal depolarization through a mechanism dependent on 2-AG and CB1 receptor activation (Marinelli et al., 2008; Yoshino et al., 2011), meaning that an increase in the expression of this enzyme could result in increased neuron excitability in PFC. Moreover, increased 2-AG signaling in the prefrontal cortex has been associated with anxiolytic-like behaviors, which are compatible with the results we observed in male offspring (Almeida-Santos et al., 2017).

Differently, female offspring of saccharin-consuming mothers showed a significant increase in the ratio Napepld/Faah; possibly indicating an increased synthesis of anandamide. Increased levels of anandamide in the PFC have been associated with increased time spent in the open arm, also in a previous study (Aliczki et al., 2016), consistent with the results we observed in the behavior of female saccharin rats. However, it is important to underline that these measures are derived from mRNA levels and do not directly reflect enzymatic activity or endocannabinoid availability, even though they still offer useful information on the relative regulation of pathways involved in endocannabinoid turnover.

### 4.4 Alterations in glutamatergic and GABAergic signaling

Alterations dependent on sex were also observed in the expression of glutamatergic and GABAergic receptors, which play crucial roles in the neurotransmission of the prefrontal cortex. In particular, male offspring of saccharin-consuming mothers presented an increase in the expression of the mRNA of various glutamatergic receptors (*Grin1, Grin2a, Grin2c, Gria1, Grm5*) compared to both control males and saccharin female rats. It is known that saccharin consumption can influence glutamate levels in the brain (Bielavska et al., 2000), but the mechanisms by which gestational saccharin can influence glutamate transmission have not been discovered or investigated. However, animal studies have shown that an increase in the expression of *Cnr2* in the prefrontal cortex is associated with an inhibition of glutamate release (Morris et al., 2021), therefore, the decrease in *Cnr2* levels could explain the increase in glutamatergic signaling observed in these animals. Glutamate receptors in the brain mediate neuronal electrical activity by regulating calcium influx, and increased activation of them may result in excitotoxic processes, culminating in neuronal damage and the development of neurological diseases (Lau and Tymianski, 2010). Moreover, NMDA receptors are essential in neuronal development, synaptic plasticity, learning, and cell survival. Dysregulation of their trafficking is implicated in neuropsychiatric disorders such as cocaine addiction, chronic alcohol abuse, schizophrenia, and Alzheimer’s disease (Lau and Zukin, 2007; Glasgow et al., 2015). On the other hand, the trafficking of AMPA receptors has been identified as a crucial regulatory mechanism in the control of synaptic plasticity, learning, and memory (Anggono and Huganir, 2012).

Differently from males, female adolescents of saccharin-consuming mothers did not show any significant variation in glutamatergic signaling but displayed a significant decrease in the expression of *Gabrg1* (GABA_*A*_R-γ1) and *Gabbr2* (GABA_*B*_R-2) genes when compared to their control littermates, denoting a marked sexual dimorphism in the effects of prenatal saccharin consumption. Proper inhibitory GABAergic signaling is essential for normal neural circuit function. If the inhibition is disturbed, excessive excitation results in a disturbance in the excitatory/inhibitory balance and, consequently, in a dysfunction of emotional and cognitive processes (Braat and Kooy, 2015). Cortico-striatal GABA pathways control motivated behavior and impulsivity, along with monoamines (Hayes et al., 2014). Disruptions in GABAergic transmission have been associated with several neurodevelopmental disorders (Braat and Kooy, 2015). GABA_*A*_ receptors can influence proliferation and migration and can inhibit cells by modulating the function of K + and Ca2 + channels (Bassetti, 2022). GABA_*A*_ silencing in the prefrontal cortex has been related to deficits in decision-making (Butkovich et al., 2015) and antagonism at this receptor can induce various cognitive, emotional, and dopaminergic abnormalities (Tse et al., 2015), including decreased attention (Paine et al., 2011). Moreover, highly impulsive rats exhibit decreased GABA_*A*_ receptor binding in the cortex (Hayes et al., 2014), thus explaining the decreased open arms latency observed in saccharin female offspring. GABA_*B*_ receptors are expressed pre- and post-synaptically, both in glutamatergic and GABAergic terminals, which is why they are capable of modulating neuronal activity, plasticity, and the balance between excitatory and inhibitory synaptic transmission in response to different concentrations of extracellular GABA (Bassetti, 2022). Similarly to GABA_*A*_, also deletion of GABA_*B*_ in the prefrontal cortex affected goal-directed behaviors (Mederos et al., 2021); reduced activity of this receptor was previously reported in the prefrontal cortex of a model of neurodevelopmental disorder (fragile X syndrome) (Kramvis et al., 2020). From this evidence, we can deduce that an altered GABAergic signal in PFC is coupled with abnormal behavioral outcomes and may justify the results observed in our study.

## 5 Conclusion

To sum up, the behavioral results observed in saccharin-exposed offspring may not indicate reduced anxiety, but rather an exaggeration of the novelty-seeking and risk-taking behaviors characteristic of adolescence. Prenatal exposure to saccharin induces neurobiological alterations in glutamatergic, GABAergic, cannabinoid, and insulin signaling pathways, which in turn affect the brain circuits responsible for modulating adolescent exploratory behavior. In this context, saccharin may disrupt the balance between fear and novelty seeking, leading offspring to exhibit a more disinhibited pattern of exploration in the open field and in the plus maze. These neurobiological and behavioral changes could be related to the development of behavioral disorders, likely due to the activation of glutamatergic signaling and the reduction of GABAergic signaling in the prefrontal cortex, a key region involved in the regulation of cognitive, behavioral, and neuroendocrine processes with sex-specific mechanisms. Further studies are needed to determine whether the behavioral and neurochemical changes observed in adolescent rats exposed to gestational saccharin persist in adulthood and older ages, which we acknowledge as a limitation of the present study.

## Data Availability

The raw data supporting the conclusions of this article will be made available by the authors, without undue reservation.
