# Supplementary material for: Long-term consequences of prenatal saccharin exposure: evidence of sex-specific molecular programing in the prefrontal cortex and behavior of adolescent rats
Source: Front Behav Neurosci. 2026 Apr 30;20:1815692. doi: 10.3389/fnbeh.2026.1815692 (PMC13171810; doi:10.3389/fnbeh.2026.1815692)

Supplementary Material

**Long-term consequences of prenatal saccharin exposure: evidence of sex-specific molecular programming in the prefrontal cortex and behavior of adolescent rats**

Beatriz Pacheco-Sánchez^# 1,2^, Raquel López-Merchán^#2^, Pablo Rubio^2^, Pilar Martos^3^, Juan Suárez^2,3^, Carlos Sanjuan^4^, Leticia Rubio^2,3^, Stella Martin-de-las-Heras^2,3^, Fernando Rodríguez de Fonseca^2,5^, Francisco Alén^6^, Marialuisa de Ceglia^1,2*^, Patricia Rivera^2,8*^

# Supplementary Figures and Tables

**Table 1:** *Taqman® Gene Expression Assay IDs.*

| Gene symbol | Assay ID | Amplicon length (bp) |
| --- | --- | --- |
| *Actb* | Rn00667869_m1 | 91 |
| *Cnr1* | Rn02758689_s1 | 92 |
| *Cnr2* | Rn01637601_m1 | 68 |
| *Napepld* | Rn01786262_m1 | 71 |
| *Faah* | Rn00577086_m1 | 63 |
| *Daglα* | Rn01454304_m1 | 67 |
| *Daglβ* | Rn01453770_m1 | 57 |
| *Mgll* | Rn00593297_m1 | 78 |
| *Gpr55* | Rn03037213_s1 | 143 |
| *Grin1* | Rn01436034_m1 | 73 |
| *Grin2a* | Rn00561341_m1 | 78 |
| *Grin2b* | Rn00680474_m1 | 90 |
| *Grin2c* | Rn00561359_m1 | 57 |
| *Gria1* | Rn00709588_m1 | 85 |
| *Gria2* | Rn00568514_m1 | 122 |
| *Gria3* | Rn00583547_m1 | 75 |
| *Gria4* | Rn00568544_m1 | 76 |
| *Grm3* | Rn01755349_m1 | 150 |
| *Grm5* | Rn00566628_m1 | 112 |
| *Gabra1* | Rn00788315_m1 | 75 |
| *Gabra2* | Rn01413643_m1 | 123 |
| *Gabrb1* | Rn00564146_m1 | 81 |
| *Gabrb2* | Rn00564149_m1 | 64 |
| *Gabrg1* | Rn00589841_m1 | 73 |
| *Gabrg2* | Rn01464079_m1 | 78 |
| *Gabbr1* | Rn00578911_m1 | 113 |
| *Gabbr2* | Rn00582550_m1 | 87 |
| *Insr* | Rn00690703_m1 | 68 |
| *Irs1* | Rn02132493_s1 | 147 |
| *Irs2* | Rn01482270_s1 | 61 |

**Table 2:** *Antibodies used for protein expression by Western blotting.*

| Antibody | Manufacturer and Catalogue Number | Host | Dilution |
| --- | --- | --- | --- |
| Adaptin | BD Biosciences 610385 | Mouse | 1/2000 |
| IRS1 | Sigma Aldrich #06-248 | Rabbit | 1/10000 |
| p-IRS1(Ser612) | Cell Signaling #3203 | Rabbit | 1/1000 |
| p-IRS1(Tyr896) | Abcam #ab46800 | Rabbit | 1/500 |
| PI3K | Cell Signaling #4257 | Rabbit | 1/1000 |
| p-PI3K-p85(Tyr607) | Abcam #ab182651 | Rabbit | 1/1000 |
| AKT | Cell Signaling #9272 | Rabbit | 1/1000 |
| p-AKT(Ser473) | Santa Cruz #sc-16646R | Rabbit | 1/1000 |
| GSK3β | Cell Signaling #12456 | Rabbit | 1/1000 |
| p-GSK3β(Ser9) | Cell Signaling #5558 | Rabbit | 1/1000 |

**Table 3:** *Two-way ANOVA details for open field test.*

|  | **Factor** | **F (df)** | **p** | **Post-hoc test** |
| --- | --- | --- | --- | --- |
| ***OF Locomotion*** | Interaction | F (1, 28) = 0,132 | p=0,719 | Tukey |
|  | Sex | F (1, 28) = 1,395 | p=0,247 |  |
|  | Treatment | F (1, 28) = 0,514 | p=0,479 |  |
| ***OF Center*** | Interaction | F (1, 28) = 0,183 | p=0,672 | Fisher |
|  | Sex | F (1, 28) = 0,267 | p=0,610 |  |
|  | Treatment | F (1, 28) = 7,644 | **p=0,010** |  |
| ***OF Periphery*** | Interaction | F (1, 28) = 1,372 | p=0,251 | Tukey |
|  | Sex | F (1, 28) = 0,251 | p=0,620 |  |
|  | Treatment | F (1, 28) = 0,212 | p=0,649 |  |
| ***Time spent in periphery (per minute)*** | Time | F (1,243, 34,79) = 1,736 | p=0,1972 | Tukey |
|  | Treatment | F (1, 28) = 0,2132 | p=0,6478 |  |
|  | Sex | F (1, 28) = 0,2510 | p=0,6203 |  |
|  | Time x Treatment | F (4, 112) = 0,4773 | p=0,7523 |  |
|  | Time x Sex | F (4, 112) = 0,2065 | p=0,9344 |  |
|  | Treatment x Sex | F (1, 28) = 1,373 | p=0,2512 |  |
|  | Time x Treatment x Sex | F (4, 112) = 1,847 | p=0,1249 |  |
| ***Time spent in center (per minute)*** | Time | F (2,425, 67,90) = 2,434 | p=0,0847 | Fisher |
|  | Treatment | F (1, 28) = 7,644 | **p=0,0100** |  |
|  | Sex | F (1, 28) = 0,2667 | p=0,6096 |  |
|  | Time x Treatment | F (4, 112) = 1,050 | p=0,3847 |  |
|  | Time x Sex | F (4, 112) = 1,886 | p=0,1179 |  |
|  | Treatment x Sex | F (1, 28) = 0,1831 | p=0,6720 |  |
|  | Time x Treatment x Sex | F (4, 112) = 0,7000 | p=0,5935 |  |

**Table 4:** *Two-way ANOVA details for elevated plus maze test.*

|  | **Factor** | **F (df)** | **p** | **Post hoc test** |
| --- | --- | --- | --- | --- |
| ***EPM Locomotion*** | Interaction | F (1, 28) = 1,276 | p=0,269 | Tukey |
|  | Sex | F (1, 28) = 1,232 | p=0,277 |  |
|  | Treatment | F (1, 28) = 2,236 | p=0,147 |  |
| ***EPM closed arms*** | Interaction | F (1, 28) = 1,392 | p=0,249 | Tukey |
|  | Sex | F (1, 28) = 0,036 | p=0,850 |  |
|  | Treatment | F (1, 28) = 0,017 | p=0,897 |  |
| ***EPM open arms*** | Interaction | F (1, 28) = 0,164 | p=0,689 | Tukey |
|  | Sex | F (1, 28) = 0,215 | p=0,647 |  |
|  | Treatment | F (1, 28) = 0,315 | p=0,580 |  |
| ***EPM total entries*** | Interaction | F (1, 28) = 2,871 | p=0,103 | Tukey |
|  | Sex | F (1, 28) = 0,379 | p=0,538 |  |
|  | Treatment | F (1, 28) = 3,637 | p=0,068 |  |
| ***EPM closed entries*** | Interaction | F (1, 28) = 1,460 | p=0,238 | Tukey |
|  | Sex | F (1, 28) = 1,066 | p=0,312 |  |
|  | Treatment | F (1, 28) = 2,433 | p=0,131 |  |
| ***EPM open entries*** | Interaction | F (1, 28) = 2,991 | p=0,096 | Tukey |
|  | Sex | F (1, 28) = 0,290 | p=0,595 |  |
|  | Treatment | F (1, 28) = 2,658 | p=0,116 |  |
| ***EPM open arms latency*** | Interaction | F (1, 28) = 3,101 | p=0,090 | Tukey |
|  | Sex | F (1, 28) = 2,419 | p=0,132 |  |
|  | Treatment | F (1, 28) = 1,567 | p=0,222 |  |

**Table 5:** *Two-way ANOVA details for gene and protein expression of the insulin pathway.*

|  | **Factor** | **F (df)** | **p** | **Post hoc test** |
| --- | --- | --- | --- | --- |
| ***Insr*** | Interaction | F (1, 28) = 1,820 | p=0,1881 | Tukey |
|  | Sex | F (1, 28) = 0,05411 | p=0,8177 |  |
|  | Treatment | F (1, 28) = 1,353 | p=0,2546 |  |
| ***Irs1*** | Interaction | F (1, 28) = 2,084 | p=0,1600 | Tukey |
|  | Sex | F (1, 28) = 1,875 | p=0,1817 |  |
|  | Treatment | F (1, 28) = 1,875 | p=0,1817 |  |
| ***Irs2*** | Interaction | F (1, 28) = 0,08813 | p=0,7688 | Tukey |
|  | Sex | F (1, 28) = 1,513 | p=0,2290 |  |
|  | Treatment | F (1, 28) = 0,01619 | p=0,8997 |  |
| **p-IRS1(Tyr896)** | Interaction | F (1, 20) = 7,236 | **p=0,0141** | Tukey |
|  | Sex | F (1, 20) = 5,588 | **p=0,0283** |  |
|  | Treatment | F (1, 20) = 0,3256 | p=0,5746 |  |
| **p-IRS1(Ser612)** | Interaction | F (1, 20) = 4,423 | **p=0,0483** | Tukey |
|  | Sex | F (1, 20) = 4,658 | **p=0,0432** |  |
|  | Treatment | F (1, 20) = 0,5176 | p=0,4802 |  |
| **total IRS1** | Interaction | F (1, 20) = 6,722 | **p=0,0174** | Tukey |
|  | Sex | F (1, 20) = 6,042 | **p=0,0232** |  |
|  | Treatment | F (1, 20) = 0,5998 | p=0,4477 |  |
| **p-PI3K(Tyr607)** | Interaction | F (1, 20) = 1,101 | p=0,3066 | Fisher |
|  | Sex | F (1, 20) = 8,109 | **p=0,0100** |  |
|  | Treatment | F (1, 20) = 1,230 | p=0,2806 |  |
| **total PI3K** | Interaction | F (1, 20) = 0,2388 | p=0,6304 | Tukey |
|  | Sex | F (1, 20) = 0,9552 | p=0,3401 |  |
|  | Treatment | F (1, 20) = 0,01493 | p=0,9040 |  |
| **p-AKT(Ser473)** | Interaction | F (1, 20) = 3,786 | p=0,0659 | Fisher |
|  | Sex | F (1, 20) = 64,41 | **p<0,0001** |  |
|  | Treatment | F (1, 20) = 10,38 | **p=0,0043** |  |
| **total AKT** | Interaction | F (1, 20) = 16,30 | **p=0,0006** | Tukey |
|  | Sex | F (1, 20) = 24,11 | **p<0,0001** |  |
|  | Treatment | F (1, 20) = 14,58 | **p=0,0011** |  |
| **p-GSK3β(Ser9)** | Interaction | F (1, 20) = 2,133 | p=0,1597 | Fisher |
|  | Sex | F (1, 20) = 2,996 | **p=0,0989** |  |
|  | Treatment | F (1, 20) = 0,2926 | p=0,5945 |  |
| **total GSK3β** | Interaction | F (1, 20) = 3,904 | p=0,0621 | Fisher |
|  | Sex | F (1, 20) = 4,819 | **p=0,0401** |  |
|  | Treatment | F (1, 20) = 1,205 | p=0,2854 |  |

**Table 6:** *Two-way ANOVA details for gene expression of the endocannabinoid system.*

|  | **Factor** | **F (df)** | **P** | **Post hoc test** |
| --- | --- | --- | --- | --- |
| ***Cnr1*** | Interaction | F (1, 28) = 1,546 | p=0,2240 | Tukey |
|  | Sex | F (1, 28) = 1,278 | p=0,2679 |  |
|  | Treatment | F (1, 28) = 0,2045 | p=0,6546 |  |
| ***Cnr2*** | Interaction | F (1, 28) = 0,02649 | p=0,8719 | Fisher |
|  | Sex | F (1, 28) = 0,1060 | p=0,7472 |  |
|  | Treatment | F (1, 28) = 6,282 | **p=0,0183** |  |
| ***Gpr55*** | Interaction | F (1, 28) = 1,029 | p=0,3190 | Fisher |
|  | Sex | F (1, 28) = 0,8013 | p=0,3783 |  |
|  | Treatment | F (1, 28) = 5,794 | **p=0,0229** |  |
| ***Dagla*** | Interaction | F (1, 28) = 3,146 | p=0,0870 | Tukey |
|  | Sex | F (1, 28) = 3,486 | p=0,0724 |  |
|  | Treatment | F (1, 28) = 2,231 | p=0,1465 |  |
| ***Daglb*** | Interaction | F (1, 28) = 13,16 | **p=0,0011** | Tukey |
|  | Sex | F (1, 28) = 3,032 | p=0,0926 |  |
|  | Treatment | F (1, 28) = 0,1895 | p=0,6667 |  |
| ***Mgll*** | Interaction | F (1, 28) = 4,860 | **p=0,0359** | Tukey |
|  | Sex | F (1, 28) = 0,7052 | p=0,4081 |  |
|  | Treatment | F (1, 28) = 0,01102 | p=0,9171 |  |
| ***Napepld*** | Interaction | F (1, 28) = 0,3736 | p=0,5460 | Tukey |
|  | Sex | F (1, 28) = 0,9091 | p=0,3485 |  |
|  | Treatment | F (1, 28) = 0,07233 | p=0,7900 |  |
| ***Faah*** | Interaction | F (1, 28) = 6,205 | **p=0,0189** | Tukey |
|  | Sex | F (1, 28) = 1,775 | p=0,1935 |  |
|  | Treatment | F (1, 28) = 0,5671 | p=0,4577 |  |
| ***Dagla/Mgll*** | Interaction | F (1, 27) = 0,05477 | p=0,8167 | Tukey |
|  | Sex | F (1, 27) = 1,028 | p=0,3195 |  |
|  | Treatment | F (1, 27) = 2,197 | p=0,1499 |  |
| ***Daglb/Mgll*** | Interaction | F (1, 27) = 0,08529 | p=0,7725 | Tukey |
|  | Sex | F (1, 27) = 1,147 | p=0,2937 |  |
|  | Treatment | F (1, 27) = 0,7676 | p=0,3887 |  |
| ***Napepld/Faah*** | Interaction | F (1, 26) = 1,940 | p=0,1755 | Tukey |
|  | Sex | F (1, 26) = 2,308 | p=0,1407 |  |
|  | Treatment | F (1, 26) = 1,158 | p=0,2917 |  |

**Table 7:** *Two-way ANOVA details for gene expression of glutamatergic transmission.*

|  | **Factor** | **F (df)** | **P** | **Post hoc test** |
| --- | --- | --- | --- | --- |
| ***Grin1*** | Interaction | F (1, 28) = 1,397 | p=0,2472 | Fisher |
|  | Sex | F (1, 28) = 2,688 | p=0,1123 |  |
|  | Treatment | F (1, 28) = 4,722 | **p=0,0384** |  |
| ***Grin2a*** | Interaction | F (1, 28) = 8,026 | **p=0,0085** | Tukey |
|  | Sex | F (1, 28) = 4,803 | **p=0,0369** |  |
|  | Treatment | F (1, 28) = 3,500 | p=0,0719 |  |
| ***Grin2b*** | Interaction | F (1, 28) = 0,001414 | p=0,9703 | Tukey |
|  | Sex | F (1, 28) = 0,06931 | p=0,7943 |  |
|  | Treatment | F (1, 28) = 0,03536 | p=0,8522 |  |
| ***Grin2c*** | Interaction | F (1, 28) = 9,053 | **p=0,0055** | Tukey |
|  | Sex | F (1, 28) = 11,51 | **p=0,0021** |  |
|  | Treatment | F (1, 28) = 1,807 | p=0,1896 |  |
| ***Gria1*** | Interaction | F (1, 28) = 11,91 | **p=0,0018** | Tukey |
|  | Sex | F (1, 28) = 2,978 | p=0,0954 |  |
|  | Treatment | F (1, 28) = 1,762 | p=0,1951 |  |
| ***Gria2*** | Interaction | F (1, 28) = 1,267e-030 | p>0,9999 | Tukey |
|  | Sex | F (1, 28) = 1,028 | p=0,3192 |  |
|  | Treatment | F (1, 28) = 1,481 | p=0,2338 |  |
| ***Gria3*** | Interaction | F (1, 28) = 0,6465 | p=0,4281 | Tukey |
|  | Sex | F (1, 28) = 2,740 | p=0,1090 |  |
|  | Treatment | F (1, 28) = 3,761 | p=0,0626 |  |
| ***Gria4*** | Interaction | F (1, 28) = 0,05145 | p=0,8222 | Fisher |
|  | Sex | F (1, 28) = 4,643 | **p=0,0399** |  |
|  | Treatment | F (1, 28) = 2,174 | p=0,1515 |  |
| ***Grm3*** | Interaction | F (1, 28) = 3,609 | p=0,0678 | Tukey |
|  | Sex | F (1, 28) = 0,04455 | p=0,8344 |  |
|  | Treatment | F (1, 28) = 1,114 | p=0,3003 |  |
| ***Grm5*** | Interaction | F (1, 28) = 12,82 | **p=0,0013** | Tukey |
|  | Sex | F (1, 28) = 2,424 | p=0,1307 |  |
|  | Treatment | F (1, 28) = 0,8727 | p=0,3582 |  |

**Table 8:** *Two-way ANOVA details for gene expression of GABAergic transmission.*

|  | **Factor** | **F (df)** | **p** | **Post hoc test** |
| --- | --- | --- | --- | --- |
| ***Gabra1*** | Interaction | F (1, 28) = 0,01420 | p=0,9060 | Tukey |
|  | Sex | F (1, 28) = 0,8344 | p=0,3688 |  |
|  | Treatment | F (1, 28) = 0,03943 | p=0,8440 |  |
| ***Gabra2*** | Interaction | F (1, 28) = 0,5676 | p=0,4575 | Tukey |
|  | Sex | F (1, 28) = 0,07704 | p=0,7834 |  |
|  | Treatment | F (1, 28) = 0,001572 | p=0,9687 |  |
| ***Gabrb1*** | Interaction | F (1, 28) = 0,5979 | p=0,4459 | Tukey |
|  | Sex | F (1, 28) = 0,09567 | p=0,7594 |  |
|  | Treatment | F (1, 28) = 0,05381 | p=0,8182 |  |
| ***Gabrb2*** | Interaction | F (1, 28) = 0,03106 | p=0,8614 | Tukey |
|  | Sex | F (1, 28) = 0,01118 | p=0,9165 |  |
|  | Treatment | F (1, 28) = 0,1006 | p=0,7534 |  |
| ***Gabrg1*** | Interaction | F (1, 28) = 5,573 | **p=0,0254** | Tukey |
|  | Sex | F (1, 28) = 26,67 | **p<0,0001** |  |
|  | Treatment | F (1, 28) = 8,278 | **p=0,0076** |  |
| ***Gabrg2*** | Interaction | F (1, 28) = 0,02260 | p=0,8816 | Tukey |
|  | Sex | F (1, 28) = 0,3616 | p=0,5525 |  |
|  | Treatment | F (1, 28) = 0,05085 | p=0,8232 |  |
| ***Gabbr1*** | Interaction | F (1, 28) = 0,6400 | p=0,4304 | Tukey |
|  | Sex | F (1, 28) = 0,006400 | p=0,9368 |  |
|  | Treatment | F (1, 28) = 0,6400 | p=0,4304 |  |
| ***Gabbr2*** | Interaction | F (1, 28) = 1,595 | p=0,2170 | Fisher |
|  | Sex | F (1, 28) = 20,02 | **p=0,0001** |  |
|  | Treatment | F (1, 28) = 15,69 | **p=0,0005** |  |

## Supplementary Figures

**Supplementary Figure 1:** *Graphical representation of the methodology of the study*


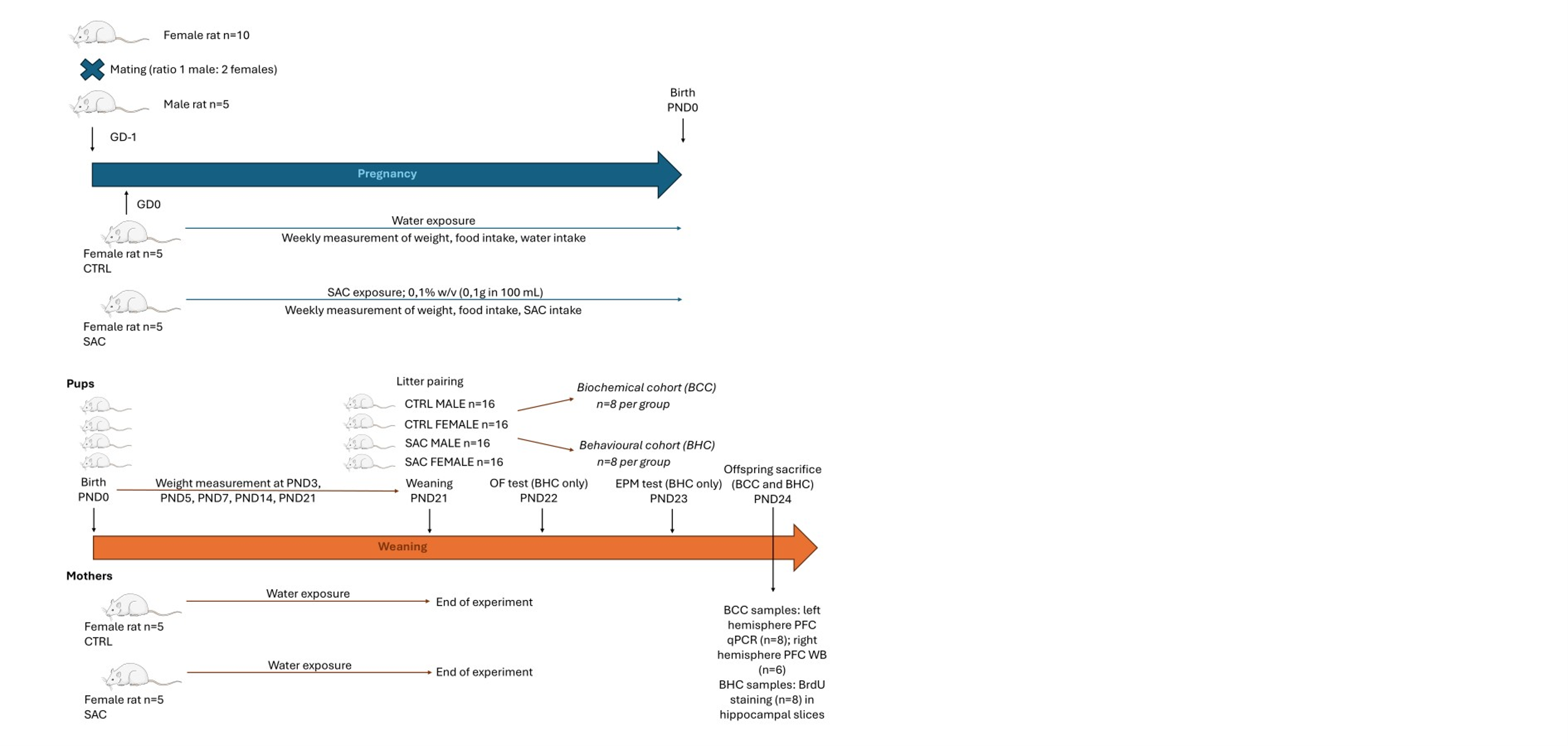


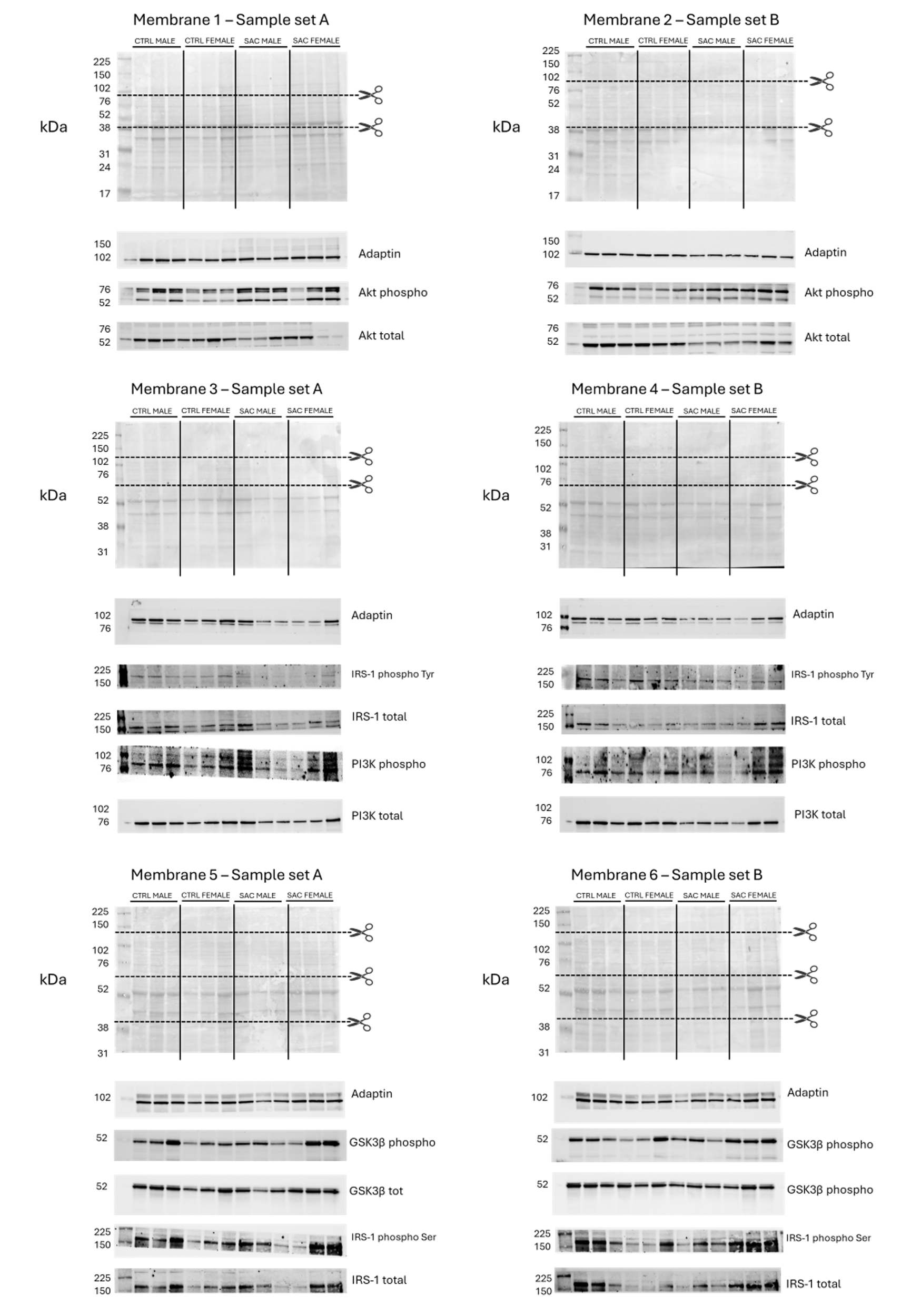
**Supplementary Figure 2:** *Ponceau Red and constitutive bands for each gel*

**Supplementary Results:**

- 1. **Dams body weight during pregnancy

     Supplementary Figure 3:** Results of Dams body weight during pregnancy

Body weight was measured in dams before pregnancy, and weekly during pregnancy. No significant variation was observed in dams body weight regardless of saccharin consumption.

- 1. **Litter size and sex ratio**

Litter sizes were 11.67±2.52 (SEM) for the control group and 11,6±4,78 for the saccharin group. Sex ratio was 42.9% males and 57.1% females in the control group and 43.1% males and 56.9% females in saccharin group.

- 1. **Pups’ weight at PND3, PND7, PND14 and PND21**

**Supplementary Figure 4:** Offspring weight at different time points after birth.


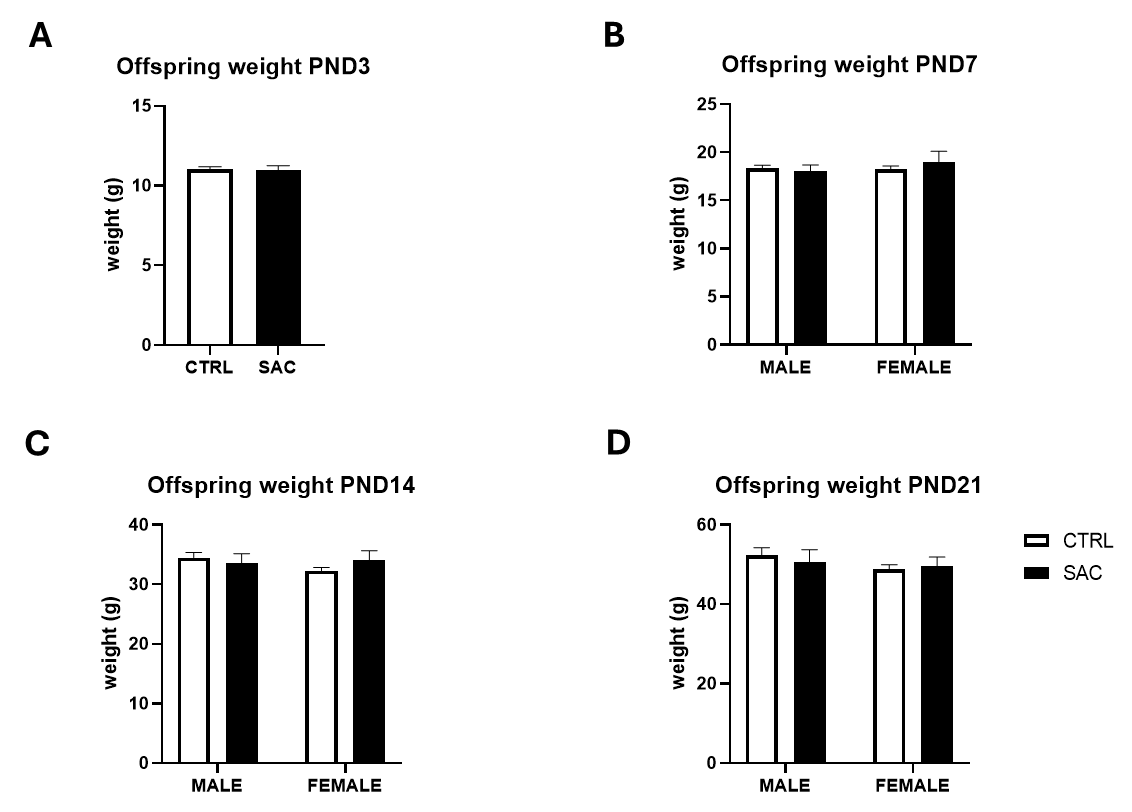


Animals were sexed on day 7. No significant difference was observed in the body weight of pups in any timepoint (One-way ANOVA (A); two-way ANOVA (B-D)).

- 1. **Study of BrdU staining in the subventricular zone of hippocampus**

Staining of BrdU in the hippocampus was made to assess the quantity of proliferating cells. BrdU was administered the day before sacrifice at a dosage of 100 mg/kg three times with a 3-hour interval between each administration. Anova shows a significant effect of treatment (p<0.05; F_1,20_=6.556) but no significant effect of sex (F=2.786, p=0.36) or interaction (F=11.72; p=0.07); significant post-hoc as shown in figure (Fisher). Female offspring of saccharine-consuming mothers showed a significant decrease in BrdU staining when compared both to female controls and saccharine males; suggesting a reduction in cell proliferation in the subgranular zone of hippocampus. n=8 for each group.

**Supplementary Figure 5:** Results of BrdU staining quantification in the hippocampus

**Supplementary Figure 6:** Significant micrographs of BrdU staining realized at 10x.
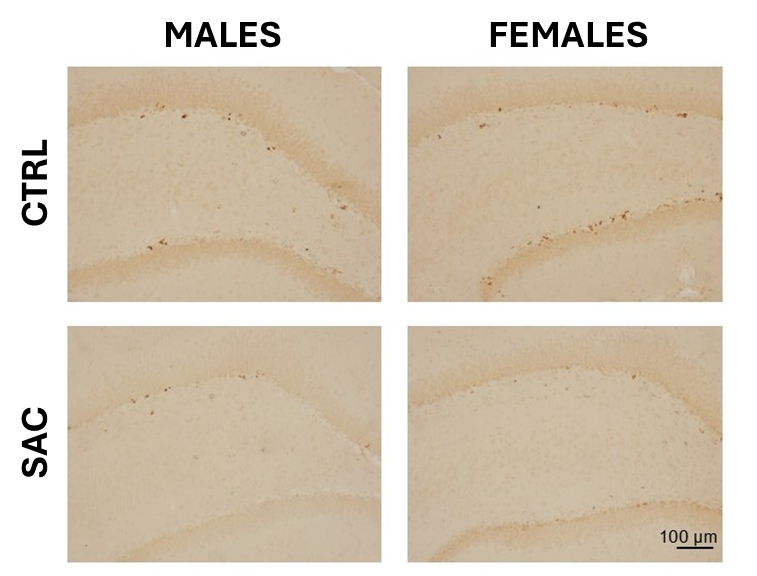

Supplement: Supplementary file 1 [file Data_Sheet_1.docx]
